# Myocardial scar detection in free-breathing Dixon-based fat- and water-separated 3D inversion recovery late-gadolinium enhancement whole heart MRI

**DOI:** 10.1007/s10554-022-02701-0

**Published:** 2022-08-23

**Authors:** Alan A. Peters, Benedikt Wagner, Giancarlo Spano, Fabian Haupt, Lukas Ebner, Karl-Philipp Kunze, Michaela Schmidt, Radhouene Neji, René Botnar, Claudia Prieto, Bernd Jung, Andreas Christe, Christoph Gräni, Adrian T. Huber

**Affiliations:** 1 Department of Diagnostic, Interventional and Pediatric Radiology, Inselspital, Bern University Hospital, University of Bern, Freiburgstrasse, 3010 Bern, Switzerland; 2 Department of Cardiology, Inselspital, Bern University Hospital, University of Bern, Bern, Switzerland; 3 MR Research Collaborations, Siemens Healthcare Limited, Frimley, UK; 4 Cardiovascular MR Predevelopment, Siemens Healthcare GmbH, Erlangen, Germany; 5 School of Biomedical Engineering and Imaging Sciences, King’s College London, St Thomas’ Hospital, London, UK

**Keywords:** Cardiac, Three-dimensional imaging, Magnetic resonance imaging, Cardiomyopathies, Pericardium, Myocardium

## Abstract

The aim of this study was to investigate the diagnostic accuracy and reader confidence for late-gadolinium enhancement (LGE) detection of a novel free-breathing, image-based navigated 3D whole-heart LGE sequence with fat–water separation, compared to a free-breathing motion-corrected 2D LGE sequence in patients with ischemic and non-ischemic cardiomyopathy. Cardiac MRI patients including the respective sequences were retrospectively included. Two independent, blinded readers rated image quality, depiction of segmental LGE and documented acquisition time, SNR, CNR and amount of LGE. Results were compared using the Friedman or the Kruskal–Wallis test. For LGE rating, a jackknife free-response receiver operating characteristic analysis was performed with a figure of merit (FOM) calculation. Forty-two patients were included, thirty-two were examined with a 1.5 T-scanner and ten patients with a 3 T-scanner. The mean acquisition time of the 2D sequence was significantly shorter compared to the 3D sequence (07:12 min vs. 09:24 min; p < 0.001). The 3D scan time was significantly shorter when performed at 3 T compared to 1.5 T (07:47 min vs. 09:50 min; p < 0.001). There were no differences regarding SNR, CNR or amount of LGE. 3D imaging had a significantly higher FOM (0.89 vs. 0.78; p < 0.001). Overall image quality ratings were similar, but 3D sequence ratings were higher for fine anatomical structures. Free-breathing motion-corrected 3D LGE with high isotropic resolution results in enhanced LGE-detection with higher confidence and better delineation of fine structures. The acquisition time for 3D imaging was longer, but may be reduced by performing on a 3 T-scanner.

## Introduction

Cardiac magnetic resonance (CMR) imaging is the gold standard for the noninvasive detection and quantification of myocardial fibrosis and scars [1, 2]. Myocardial scars, as quantified by late gadolinium enhancement (LGE), have been associated with arrhythmias and stroke [3, 4], as well as decreased long-term survival [5–7]. It is therefore important to detect and quantify myocardial scars with high accuracy and confidence.

In traditional 2D LGE sequences, a single image slice is acquired over a long breath-hold. To limit acquisition time with multiple breath-holds (one per acquired slice), inter-slice gaps can be used, resulting in incomplete coverage of the left ventricle (LV) [8, 9]. Furthermore, 2D sequences may suffer from slice misregistration due to different breath-hold positions and artifacts due to respiratory motion [10]. Even if the spatial resolution has improved with in-plane resolutions of < 2 mm, a through-plane resolution of 6–8 mm still results in partial volume artifacts limiting the depiction of small areas of LGE.

Newly developed 3D LGE sequences have an isotropic resolution in all directions and long breath-holds are no longer needed, thanks to the use of diaphragmatic navigator-based gating for respiratory motion compensation [11–15]. More recently, image navigation (iNAV) with direct respiratory motion tracking of the heart has been introduced and allows for acquiring images during the whole respiratory cycle with shorter acquisition times [16]. The isotropic high-resolution, whole-heart coverage promises a higher accuracy for detecting small areas of LGE, as well as LGE in thin structures such as the atria or the pericardium.

Another difficulty of LGE imaging is the similar signal intensity of epicardial LGE, epicardial fat and pericardial LGE [17]. Dixon-based fat-/water-separation techniques have been proposed for improved fat suppression compared to conventional fat saturated LGE [18]. Promising first results have been shown in breath-hold 2D LGE imaging [19, 20] and single breath-hold 3D LGE imaging [21, 22]. Recently, a free-breathing Dixon-based fat- and water-separated 3D inversion recovery late-gadolinium enhancement sequence with isotropic high-resolution and iNAV-based non-rigid respiratory motion correction has shown promising first results in small proof of concept studies [23, 24]. However, diagnostic accuracy and reader confidence for LGE detection have not yet been analyzed. Additionally, latest-generation 2D LGE sequences allow high-quality free-breathing acquisitions with full LV-coverage in a short scan time but have never been compared with iNAV-based 3D sequences [25, 26].

This study aimed to investigate the diagnostic accuracy and reader confidence for LGE detection of a novel free-breathing, iNAV-based 3D LGE sequence with fat–water separation on both, 1.5 T and 3 T, in comparison to a free-breathing 2D LGE sequence in patients with ischemic and non-ischemic cardiomyopathy.

## Materials/methods

### Patient population

All patients undergoing cardiac MRI between 07/2020 and 01/2021 were included. Patients without written informed consent and those younger than 18 years of age were excluded.

This retrospective study was conducted in accordance with the Declaration of Helsinki (including the later amendments) and was approved by the local institutional review board. The authors have full access and take full responsibility for the integrity of all data. Baseline clinical parameters including detailed medical history and blood tests were analyzed for all patients.

### Cardiac MRI protocol

All patients were examined on a Siemens MAGNETOM Aera 1.5 T-scanner (n = 32) and a Siemens MAGNETOM Skyra 3 T-scanner (n = 10) (both Siemens Healthineers, Erlangen, Germany). Standard cardiac MRI sequences were performed in end-expiratory breath-hold, including steady-state free precession (bSSFP) cine images in short-axis, two-, three- and four chamber views.

The 3D inversion recovery (IR) prepared spoiled gradient echo prototype sequence with isotropic high-resolution of 1.3 mm and whole-heart coverage was performed in free-breathing, covering the whole heart in transverse orientation ten minutes after intravenous injection of 0.2 mmol/kg gadobutrol (Gadovist, Bayer Schering Pharma, Zürich, Switzerland). The acquisition was performed with an undersampled variable density Cartesian acquisition with spiral-like order [27, 28]. For respiratory tracking, coronal 2D iNAV images were acquired in each heartbeat before 3D data acquisition and used for motion correction in a non-rigid motion-corrected iterative SENSE reconstruction [29]. Fat–water separation was achieved with a Dixon in- and opposed phase inversion recovery pulse every other RR interval with the following parameters: FoV of 312 × 312 mm, matrix of 240 × 240, repetition time/echo time of 4.4/2.4 ms, isotropic resolution of 1.3 mm × 1.3 mm, slice thickness: 1.3 mm. The inversion time (TI) for the myocardium was determined on a Look-Locker compressed sensing TI scout triggered on every heartbeat.

For comparison, 2D free-breathing LGE images with full left ventricular short axis coverage and 8 mm slice thickness were acquired with motion correction and eight averages [26]. This sequence consisted of a T1-weighted (T1w) fast gradient-echo (GRE) phase-sensitive inversion recovery (PSIR) sequence with inversion pulses every second RR interval with the following parameters: FoV of 276 × 340 mm, matrix of 156 × 256, repetition time/echo time 2.3/1.05 ms, in-plane resolution of 1.4 × 1.4 mm, slice thickness of 8 mm, interslice gap of 2 mm, flip angle of 25°. The inversion times of the sequences were determined before the image acquisition, by using a Look-Locker scout sequence, as described before [23].

### Image reformatting

2D LGE images were arranged on a monitor and presented as 4-chamber view, 2-chamber view and a short axis stack with the magnitude images, as well as a short axis stack with the phase-sensitive inversion recovery (PSIR) images. For optimal comparability, 3D LGE Dixon in-phase and water images were shown in 1.3 mm isovoxel resolution, reformatted in an analogous way, in a 4-chamber view, 2-chamber view and as a short axis stack, and arranged on a separate monitor.

### Reading

The readout was performed on a PACS-workstation (Sectra PACS IDS7, Sectra) with dedicated monitors (BARCO Coronis Fusion 6MP LED, Kortrijk, Belgium). All images were anonymized. Individualized readout-sheets were generated for two independent blinded readers with randomization of the patients and LGE sequences to ensure that they did not read the 2D and 3D sequences simultaneously. The readers were allowed to adjust window settings and to use multiplanar reconstruction (MPR) for the analysis of the 3D isotropic images as they would in a standard clinical readout, but did not see the other standard cardiac MRI sequences.

### Image quality

Both readers independently rated the image quality based on a five-point Likert scale. The overall image quality was rated as follows: 5—excellent (no artifacts, interpretation unaffected), 4—good (minimal artifacts, interpretation unaffected), 3—acceptable (some artifacts, interpretation slightly affected), 2—poor (heavy artifacts, interpretation significantly affected, e.g. epicardial border identified but not diagnostic for LGE detection), 1—non-diagnostic (uninterpretable). In case of poor or non-diagnostic image quality, the reasons were documented separately (e.g. motion artifacts/blurring, folding artifacts, low contrast/high noise, or inadequate myocardial nulling).

To assess the quality of anatomical details, the visibility of thin anatomical structures was additionally rated for the LV endocardial border, papillary muscles, epicardial border, as well as RV, atria and pericardial border as follows: 5—no motion, contour high resolution, aspect sharp high-resolution, 4—no motion, contour high resolution, aspect smooth high-resolution, 3—no motion, contour low resolution with partial volume, 2—significantly blurred, contour identified, 1—heavily blurred, not diagnostic.

First, the 2D LGE sequence and the 3D LGE in-phase acquisition were compared, to analyze the effect of the higher through-plane resolution of 3D LGE sequence. Secondly, the water- acquisition was compared with both the 2D LGE sequence and the 3D in-phase to assess the additional benefit of the fat/water separation.

### Segmental LGE detection and confidence rating

LGE presence or absence was rated separately for each of the 17 AHA LV-segments, as well as the pericardium, right ventricle and the atria [30]. For LV LGE, transmurality and distribution were documented. For LGE positive cases, the level of confidence was rated individually for every segment as follows: 5—highest confidence for small millimetric-sized spots of LGE, 4—high confidence for foci < 5 mm, 3—acceptable confidence for foci > 5 mm, 2—low confidence, only for foci > 10 mm, 1—no confidence even for large LGE foci.

### Quantitative LGE evaluation

Both readers quantified the amount of LGE with more than two weeks between the quantitative and qualitative readout by using a dedicated commercially available software (Circle Cardiovascular Imaging Inc., Calgary, Canada). For the LGE quantification, the 3D LGE Dixon images (water-only) were prepared as 8 mm thick short axis reconstructions to match with the respective 2D LGE-sequences. The endocardial and epicardial contours were manually segmented on all images and a region of interest was placed in a representative LGE lesion and in the remote myocardium for quantification of the LGE mass in gram (g) based on a full-width half-maximum algorithm (FWHM) segmentation of the whole LV myocardium.

### CNR/SNR

To compare contrast-to-noise ratios (CNR) and signal-to-noise ratios (SNR), the signal intensities of different regions of interest (air, myocardium, LGE) were recorded to calculate CNR and SNR using the following formulas [31]:

$$CNR\hspace{0.17em}=\frac{\mathrm{C }(Contrast\; between \;LGE \;and \;remote \;myocardium) }{N (standard\; deviation \;of \;blood \;pool \;signal)}$$

$$SNR=\frac{\mathrm{S }(signal\; of \;LGE) }{N (standard \;deviation \;of \;blood \;pool \;signal)}$$

### Statistical analysis

All analyses were performed using a dedicated statistics software (IBM SPSS Statistics, release 25.0; SPSS, Armonk, NY). P-values < 0.05 were considered statistically significant. Normal distribution was tested by Shapiro–Wilk test and non-parametric tests were used in case of non-normal distribution. Likert scales and quantitative parameters of the three sequences (2D, 3D water, 3D in-phase) were compared using Friedman test with post-hoc group comparison and Bonferroni correction. Comparisons between two groups (i.e. 1.5 T vs. 3 T) were performed using the Mann–Whitney-U-test. Inter-reader agreement was evaluated using Cohen’s kappa value (κ). κ was interpreted as follows: slight agreement (0 < κ ≤ 0.2), fair agreement (0.2 < κ ≤ 0.4), moderate agreement (0.4 < κ ≤ 0.6), substantial agreement (0.6 < κ ≤ 0.8), almost perfect agreement (0.8 < κ ≤ 1.0) [32]. For the segmental LGE detection and confidence rating, a jackknife alternative free-response receiver operating characteristic (JAFROC) analysis with fixed readers and random cases was performed. The JAFROC figure of merit (FOM) was calculated as the area under the alternative free-response receiver operating characteristic (AFROC) curve [33].

## Results

### Patient population

Forty-seven patients were included in this study. 3D LGE images were not interpretable in five patients due to arrhythmia and delayed triggering (n = 4) or severe motion artifacts (n = 1). Those patients were therefore excluded from further analyses. 2D LGE images were not acquired in one patient due to scan time restrictions. From the finally included forty-two patients, twenty-nine (69%) were males, the mean age was 52 years and the mean BMI was 24.9 kg/m^2^. The etiologies of cardiomyopathies (CMP) are shown in Table 1; while 33% of the patients suffered of ischemic cardiomyopathy (ICM), 29% were diagnosed with peri-/myocarditis and 36% with non-ischemic cardiomyopathy (NICM). Patient characteristics are presented in Table 1 as mean values with standard deviation or as relative proportions, respectively.

**Table 1:** Patient characteristics

Patient characteristics (n = 42)	
Sex (m/f)	29/13
Age (years)	51.6 ± 18.8
Height (m)	1.72 ± 0.1
Weight (kg)	74.1 ± 17.5
BMI (kg/m^2^)	24.9 ± 4.9
Creatinine (μmol/l)	94.2 ± 54.4
Heart rate (bpm)	69.9 ± 12.0
Ischemic cardiomyopathy	14/42 (33%)
Non-ischemic cardiomyopathy	15/42 (36%)
Peri-/myocarditis	12/42 (29%)

### Sequence acquisition time

The mean sequence acquisition times of the 2D LGE sequences were significantly shorter than the 3D LGE scan times (07:29 ± 02:14 min vs. 09:50 ± 03:01 min at 1.5 T and 06:12 ± 01:01 min vs. 07:47 ± 01:27 min at 3 T). When including sequence planning, TI-scout and resting-phase test scan, the entire duration of the scan protocol remained significantly shorter for 2D LGE, compared to the 3D LGE (2D: 10:54 ± 03:18 min, 3D: 13:30 ± 03:24 min; p = 0.001).

### Image quality assessment

**Fig. 1:**
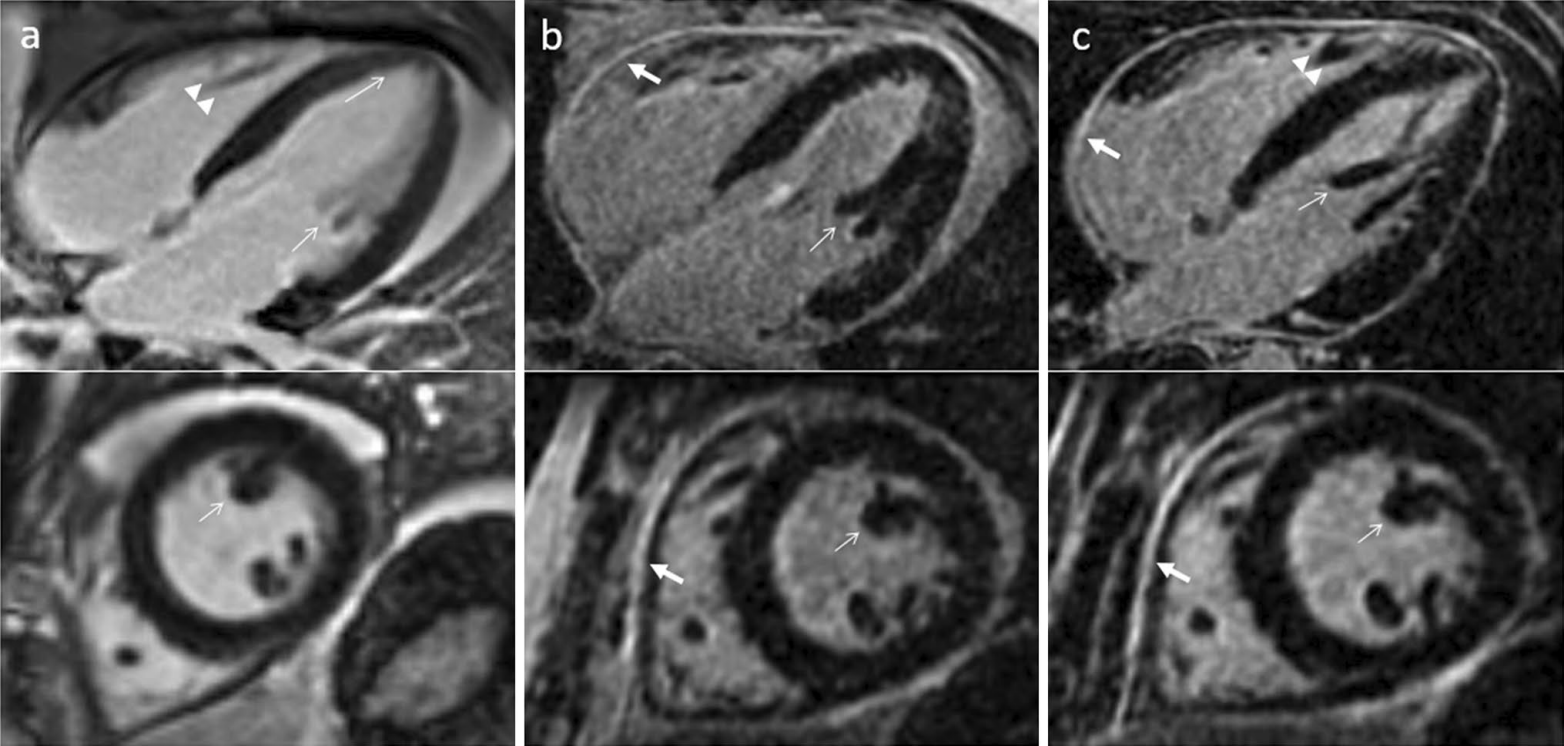
Comparison of 2D magnitude (column **a**) and 3D Dixon in-phase LGE (column **b**) imaging of the heart in 4-chamber (top row) and short axis (bottom row) views scanned at 1.5 T. Additionally, the corresponding 3D Dixon water-only acquisitions are depicted for comparison (column **c**). 3D Dixon imaging allows excellent delineation of the thin pericardium against the epicardial fat (bold arrows), while the pericardium is difficult to separate from the surrounding fat tissue in the 2D images. Also note the excellent depiction of the left ventricular papillary muscles (thin arrows) and RV trabeculae (arrowheads) in the 3D images, which are blurred in the 2D images

**Fig. 2:**
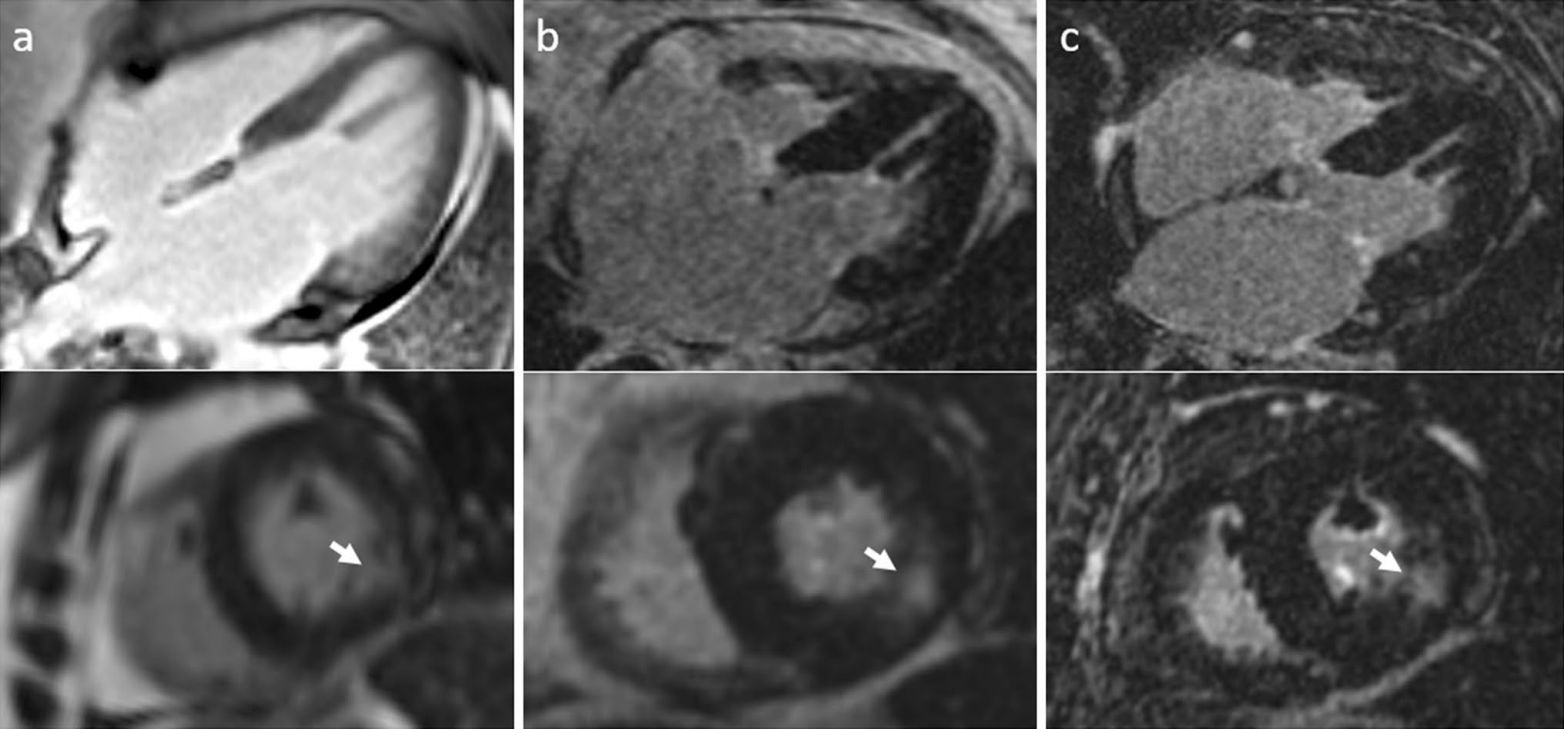
2D magnitude imaging (column **a**), 3D Dixon LGE in-phase imaging (column **b**) and 3D Dixon LGE water imaging (column **c**) showing 4-chamber (top row) and short axis (bottom row) views of a patient with subendocardial, nearly transmural myocardial LGE inferolateral in the basal and midventricular LV wall (arrows) compatible with an ischemic scar following myocardial infarction scanned at 1.5 T

**Fig. 3:**
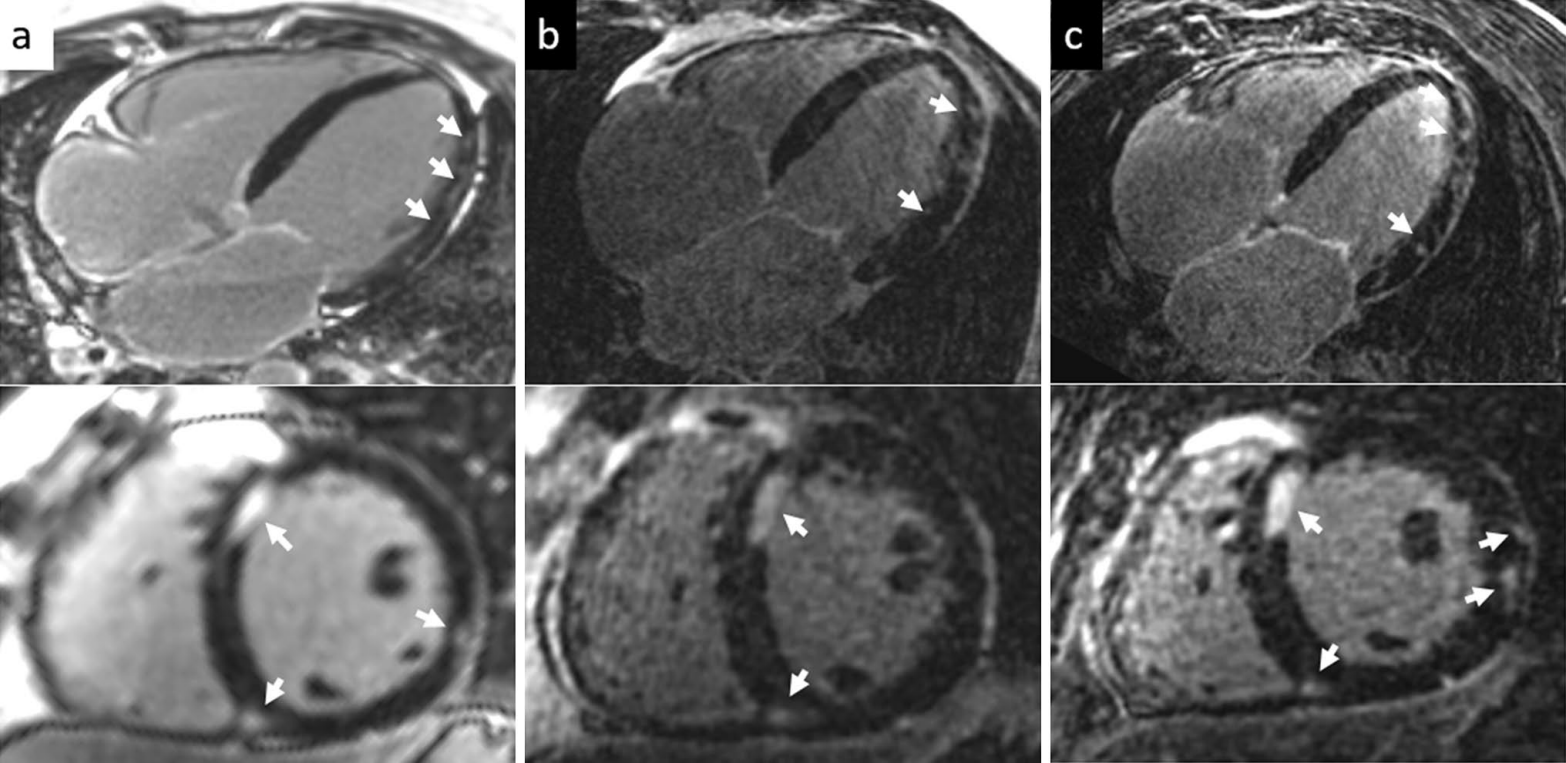
2D magnitude (column **a**), 3D Dixon in-phase (column **b**) and 3D Dixon LGE water-only (column **c**) imaging of a patient with DCM and suspected cardiac sarcoidosis scanned in short axis and 4-chamber views at 3 T with diffuse myocardial LGE in the septum close to the anterior RV insertion point and in the lateral LV wall depicted in all acquisitions (arrows)

**Fig. 4:**
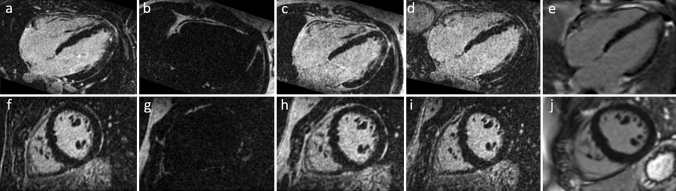
3D Dixon LGE MR imaging of a healthy patient at 1.5 T showing water-only (**a**, **f**), fat-only (**b**, **g**), in-phase (**c**, **h**), opposed phase (**d**, **i**) acquisitions and the 2D magnitude images (**e**, **j**) in 4-chamber (top row) and short axis views (bottom row)

Overall image quality was not significantly different between the 2D and 3D LGE sequences (mean image quality 3.8 for the 2D LGE sequences and 3.7 for the 3D LGE sequence; p = 0.30), as shown in Table 2. However, image quality ratings were significantly higher for the 3D LGE water acquisition for the detailed anatomical structures of the LV endocardial border, the papillary muscles, RV border and pericardium, as compared with the 2D LGE sequence, with the exception of the atria and the epicardial border. When directly comparing the 3D water and 3D in-phase acquisitions, the image quality ratings were better for the 3D water acquisitions atria and the pericardium. Results of the post-hoc analysis are shown in Table 3. Exemplary images are depicted in Figs. 1, 2, 3,4.

**Table 2:** Mean quality ratings of overall image quality and detailed anatomical structures of the 2D, 3D in-phase and 3D water acquisitions

Image quality assessment	2D	3D (in-phase)	3D (water)	*p*-value^a^
Overall image quality	3.8 ± 0.5	3.7 ± 0.7	3.7 ± 0.7	0.480
LV endocardial border	3.9 ± 0.5	4.0 ± 0.8	4.1 ± 0.8	0.024*
LV papillary muscles	3.9 ± 0.5	4.0 ± 0.8	4.1 ± 0.8	0.025*
RV border	3.6 ± 0.5	3.5 ± 0.9	3.8 ± 0.9	0.010*
Atria	3.6 ± 0.4	3.6 ± 0.8	3.8 ± 0.8	0.021*
Epicardial border	3.8 ± 0.4	3.9 ± 0.8	4.0 ± 0.8	0.142
Pericardium	3.7 ± 0.5	3.7 ± 0.8	4.0 ± 0.8	0.017*

*Significant in post-hoc analysis

^a^Friedman test

**Table 3:** Post-hoc analysis of the three-way comparison

Post-hoc analysis (p-values)	2D vs. 3D in-phase	2D vs. 3D water	3D in-phase vs. 3D water
LV endocardial border	0.663	0.019	0.56
LV papillary muscles	0.548	0.019	0.081
RV border	0.513	0.009	0.05
Atria	0.827	0.44	0.025
Epicardial border	0.676	0.132	0.312
Pericardium	0.785	0.019	0.038

### Performance and confidence of LGE detection

Readers detected segmental LGE more accurately with a higher confidence in 3D imaging, as the JAFROC analysis revealed a significantly higher figure of merit (FOM) for the 3D acquisitions (FOM, 0.89) compared to the 2D LGE sequence (FOM, 0.78; p < 0.001). The stand-alone 3D water acquisition was significantly higher than the 2D sequence a well (FOM, 0.86; p = 0.007). Examples of LGE imaging are depicted in Figs. 1, 2 and 3, the results of the JAFROC analysis are shown in Table 4.

**Table 4:** Performance and confidence of LGE detection with JAFROC figure of merit (FOM) comparison

LGE detection	FOM	p-value vs. 2D	p-value vs. 3D (water)
2D	0.78	–	0.007
3D (combined)	0.89	< 0.001	0.400
3D (water)	0.86	0.007	–

### LGE quantification and interrater variability

The total number of LGE-positive segments was 176, with a mean number of LGE-positive segments per patient of 5.8. Thirty-four lesions (19.4%) were endocardial, 107 (mid-) myocardial (61.1%) and thirty-five lesions subepicardial (20.0%). There was no statistical difference between 3D and 2D LGE sequence in the quantified amount of LGE (9.6 g vs. 11.1 g, p = 0.22), regarding myocardial LGE pattern (p = 0.1), transmurality (p = 0.09), or pericardial LGE pattern (p = 0.18).

There was substantial interrater-agreement between the two readers regarding the myocardial LGE pattern in the 2D sequence (κ = 0.66) and moderate agreement in the 3D LGE sequence (κ = 0.54). The interrater agreement between the two readers regarding the image quality scores was fair (κ = 0.25 for the 2D sequence, κ = 0.37 for the 3D sequence).

### Comparison of sequence acquisition time, SNR and CNR between 1.5 T and 3 T

Thirty-two CMR examinations were performed on a 1.5 T-scanner and 10 examinations on a 3 T-scanner. Scan duration of the 3D LGE sequence was significantly shorter on the 3 T-scanner, but there were no significant differences in image quality, SNR or CNR. In 2D LGE imaging, there was no significant difference in scan duration between 1.5 T and 3 T (Table 5).

**Table 5:** Field strength comparison regarding image quality, SNR and CNR in 2D and 3D imaging

Field strength comparison	1.5 T (n = 32)	3 T (n = 10)	*p*-value^a^
*2D magnitude imaging*			
Sequence time (min:s)	07:29 ± 02:14	06:12 ± 01:01	0.099
Overall image quality	3.8 ± 0.5	3.9 ± 0.4	0.759
SNR LGE	32.6 ± 17.0	32.6 ± 24.6	0.715
CNR LGE—myocardium	20.3 ± 18.9	20.2 ± 24.4	0.658
*3D Dixon imaging*			
Sequence time (min:s)	09:50 ± 03:01	07:47 ± 01:27	0.045
Overall image quality	4.0 ± 0.6	3.8 ± 0.6	0.318
SNR LGE	17.0 ± 9.7	16.9 ± 8.1	0.705
CNR LGE-myocardium	8.8 ± 9.9	8.8 ± 8.7	0.906

^a^Mann–Whitney-U-test

## Discussion

This study compared the diagnostic accuracy and reader confidence for LGE detection between a free-breathing motion-corrected 2D LGE sequence without fat–water separation and a free-breathing motion-corrected 3D LGE sequence with fat–water separation. The overall image quality ratings, LGE quantification, transmurality and distribution analysis were not significantly different between 2 and 3D imaging, which is consistent with earlier studies [23, 24, 34]. Furthermore, the subjective quality ratings of the 2D sequence and the non-fat saturated and therefore comparable 3D in-phase images were not significantly different. However, readers had a higher confidence in the 3D LGE images for accurate detection of LGE foci, which was revealed by the JAFROC analysis. This finding can be explained by the 1.3 mm isotropic resolution of the 3D LGE sequence that allows for detection and localization of LGE with higher confidence. One important note is that even if the technical in-plane resolution between the 2D (1.4 mm) and 3D (1.3 mm) sequence is similar, the large slice thickness of 8 mm of the 2D sequence leads to a partial volume effect in structures that are smaller than 8 mm, such as the papillary muscles or small amount of LGE. Therefore the borders of small structures such as papillary muscles or small amount of LGE are sharper in the 3D sequence, due to the smaller partial volume effects.

Due to its higher spatial resolution, the 3D LGE sequence was also more accurate for visualization of delicate anatomical structures such as the atria and the pericardium. For the epicardial borders, there was only a non-significant trend toward higher quality ratings with 3D imaging, which can be explained by the fat-/water-separation, resulting in a similar signal intensity between the nulled myocardium and the suppressed epicardial fat. However, even if the fat suppression of epicardial fat does not improve delineation of remote myocardium in comparison to epicardial fat, it enhances the delineation of small epicardial LGE-lesions adjacent to the surrounding fat tissue and increases the diagnostic accuracy and reader confidence for LGE detection in this area.

A challenge of 3D LGE imaging is the lower SNR of the 3D LGE sequences that is explained by the smaller voxel size with isotropic 1.3 mm resolution, as well as the contrast washout during the longer scan duration with consecutive TI alteration [35]. Keegan et al. proposed dynamic TI to target this issue in an earlier publication [36]. Additionally, the 2D sequence was performed on every second RR interval, allowing for a longer magnetization recovery compared to the 3D sequence that was performed on every RR interval. Five examinations had to be excluded due to arrhythmia and delayed triggering or severe motion artifacts, which is similar to the aforementioned earlier publications [23, 24].

The 3D sequence showed a technical failure rate of 10.6% (n = 5/47). This occurred mainly in patients with arrythmia or patient motion during the scan. In patients with arrhythmia, the 3D sequence may be triggered to the systole as the resting phase and not the diastole for more robust results. Nevertheless, the acquisition of the free-breathing 3D LGE sequence in patients with arrhythmia and motion during the scan remains a challenge and the 2D LGE sequence may be more robust in those cases. Further studies focusing more on the acquisition of the 3D LGE sequence in patients with arrhythmia and triggering on the systole are therefore warranted.

The significantly longer scan duration of 3D LGE imaging compared to 2D LGE imaging has been decreased over the years. Back in 2015, Andreu et al. reported mean scan times of 16 ± 7.19 min for earlier 3D LGE sequences [35]. Due to technical improvements such as image navigators (iNAV) [23], 100% respiratory scan efficiency and predictable scan times can be achieved, the latter relies solely on the patient’s heart rate and is no longer altered by changes in the respiration pattern. The mean sequence acquisition time of 09:23 min for the 3D LGE sequence in the current study is therefore much faster, but still significantly longer than the 07:12 min for the free-breathing motion-corrected 2D LGE sequence; however, the latter has a significantly lower spatial resolution in slice direction (3D: 1.3 mm, 2D: 8 mm). Acquisition times are similar to the recently reported scan times ranging between 08:00 and 10:46 min for 3D LGE sequences and between 05:36 and 09:32 min for 2D LGE sequences, depending on the acquired views and number of slices [23, 24]. In the subgroup of patients undergoing the 3D LGE scan on a 3 T-scanner, sequence acquisition times were significantly shorter than on the 1.5 T-scanner with a mean acquisition time of 07:47 min.

This study has several limitations. First, there was a relatively small sample size for the subgroup comparison between the 1.5 T- and the 3 T-scanner. For a valid conclusion, the comparison of acquisition times and image quality compared between 1.5 T and 3 T should therefore be validated in a larger patient population. Then, the comparison between 3D LGE imaging without PSIR and the 2D LGE imaging with and without PSIR might have increased the 2D LGE quality rating and would be lower, if only the 2D LGE magnitude images had been shown. However, this is more accurate for a real reading setting, where usually both, magnitude and PSIR images are analyzed in combination. In a subsequent study, a 3D LGE PSIR sequence should be investigated as well [37]. Another fact limiting the comparability is that the sequences had slightly different in-plane resolutions (1.3 vs. 1.4 mm). As the 2D-LGE sequence was optimized for a 1.4 mm in-plane resolution and an 8 mm through-plane resolution, we decided not to change any parameters on the free-breathing 2D-LGE sequence as the reference standard. A reduction of the in-plane and trough-plane resolution of the 2D-LGE sequence would significantly increase the scan duration and may result in a significant reduction of the signal-to-noise and contrast-to-noise ratio. Another limitation is the sequential acquisition of the 2D sequence after the 3D sequence. This may have introduced a bias due to contrast washout between the 3D and 2D sequence in the present study. The final limitation is the retrospective character of the analysis and the relative heterogeneity of the underlying etiology of ischemic and non-ischemic cardiomyopathies. Further investigations should focus on the detectability of small LGE lesions in subepicardial and pericardial distribution with increased sensitivity due to the fat-/water-separation, for example in patients with myocarditis and sarcoidosis.

In conclusion, free-breathing motion-corrected 3D LGE with high isotropic resolution results in better LGE-detection with higher confidence and enhanced delineation of fine anatomical structures such as the atria or pericardium. The scan acquisition time for the 3D LGE sequence was slightly longer than the 2D LGE sequence but may be reduced when performing the sequence on a 3 T-scanner.

## Abbreviations

2D: Two-dimensional
3D: Three-dimensional
AFROC: Alternative free-response receiver operating characteristic
ARVD: Arrhythmogenic right ventricular dysplasia
CMR: Cardiac magnetic resonance
EF: Ejection fraction
GRE: Gradient echo
ICM: Ischemic cardiomyopathy
JAFROC: Jackknife alternative free-response receiver operating characteristic
TI: Inversion time
LA: Left atrium
LV: Left ventricle
LGE: Late gadolinium enhancement
ms: Milliseconds
MPR: Multiplanar reconstruction
MRI: Magnetic resonance imaging
NICM: Non-ischemic cardiomyopathy
NYHA: New York heart association
RA: Right atrium
ROC: Receiver operating characteristic
RV: Right ventricle
SSFP: Steady-state free precession
SV: Stroke volume
T: Tesla
